# Type 2 Diabetes Mellitus Modifies the Prognostic Value of LDL Particle Size in Statin‐treated Patients With Coronary Artery Disease: A Large‐scale Cohort Study

**DOI:** 10.1002/mco2.70919

**Published:** 2026-08-26

**Authors:** Xiaohui Bian, Jining He, Chenxi Song, Rui Zhang, Sheng Yuan, Guofeng Gao, Kefei Dou

**Affiliations:** ^1^ State Key Laboratory of Cardiovascular Disease Beijing China; ^2^ Cardiometabolic Medicine Center Fuwai Hospital National Center for Cardiovascular Diseases Chinese Academy of Medical Sciences and Peking Union Medical College Beijing China; ^3^ Department of Cardiology Fuwai Hospital National Center for Cardiovascular Diseases Chinese Academy of Medical Sciences and Peking Union Medical College Beijing China; ^4^ National Clinical Research Center for Cardiovascular Diseases Beijing China

**Keywords:** coronary artery disease, LDL particle size, LDL‐C/ApoB ratio, risk prediction, type 2 diabetes mellitus

## Abstract

LDL‐cholesterol (LDLC) to apolipoprotein B (ApoB) ratio is an accessible and reliable proxy for the LDL‐particle size. The aim of the present study was to investigate whether type 2 diabetes mellitus (T2DM) modulates the prognostic value of LDL‐C/ApoB ratio in statin‐treated patients with coronary artery disease (CAD). The study included 21,382 patients with CAD who had received statin therapy at baseline with a median follow‐up of 3.1 years. The primary endpoint was major adverse cardiac events (MACE), including all‐cause mortality, non‐fatal myocardial infarction (MI), and ischemia‐driven revascularization. Higher LDL‐C/ApoB level (≥1.2) was associated with a decreased risk of MACE in the T2DM group (adjusted HR: 0.81, 95% CI: 0.69–0.96). A stepwise increase of 0.1 unit in LDL‐C/ApoB ratio was associated with a 5% decrease for MACE only in the T2DM group. Among participants without T2DM, there was no clear association between LDL‐C/ApoB and MACE (*p* for interaction < 0.05). No significant association was identified between MACE risk and LDL‐C or ApoB in the T2DM group. Our data demonstrated that in CAD patients who had received statin therapy, LDL‐C/ApoB ratio was related to adverse prognosis only in those with T2DM. Among patients with CAD and T2DM who are receiving statin treatment, LDL‐C/ApoB may be a better indicator of residual cholesterol risk than LDL‐C.

## Graphical abstract

The LDL‐C/ApoB ratio, a marker of LDL size, showed significant prognostic value in statin‐treated CAD patients depending on diabetes status. A higher ratio was linked to lower cardiovascular risk only in patients with type 2 diabetes, suggesting it may be a superior biomarker for risk stratification beyond conventional lipids in this high‐risk group.

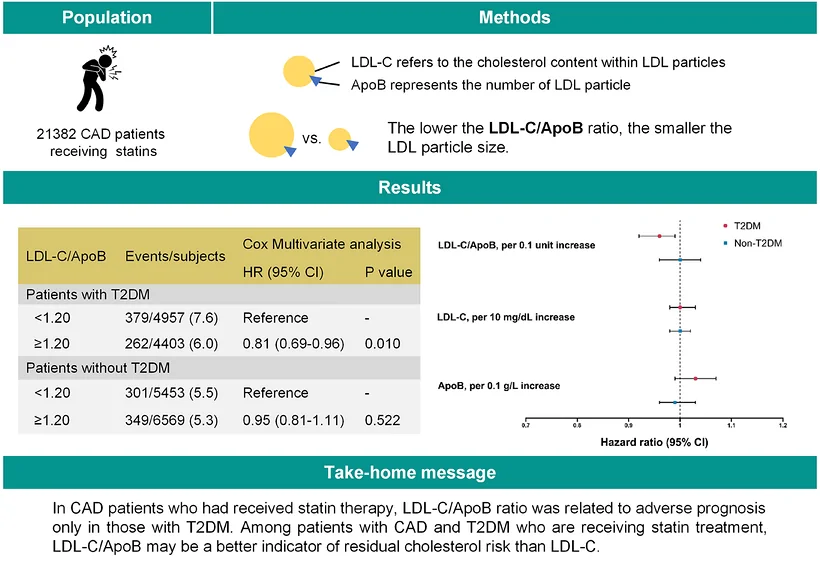

## 1 Introduction

Low‐density lipoprotein (LDL) has been proven to be the causal factor of atherosclerotic cardiovascular disease (ASCVD) [1, 2]. In addition to cholesterol content, the atherogenic effect of LDL particles also depends on their size and density [3, 4]. Compared to large, buoyant LDL (lbLDL) particles, small, dense LDL (sdLDL) particles are more atherogenic due to their greater penetration into the arterial wall, longer plasma half‐life time, lower affinity for hepatic LDL receptor, and higher susceptibility to oxidation and glycation [5, 6].

The size of LDL particles could be measured by nuclear magnetic resonance spectroscopy, density gradient ultracentrifugation, and nondenaturing gradient‐gel electrophoresis (GGE) [7, 8], which are too complex and high‐cost to promote in routine clinical practice. The ratio of LDL‐C and apolipoprotein B (ApoB) could be used as a simple and practical method to estimate the size of LDL particles, which was significantly correlated with LDL particle size determined by GGE [9]. Each LDL particle carries only one molecule of ApoB, and LDL particles account for over 90% of plasma ApoB‐containing particles, which means that plasma ApoB concentration roughly indicates the number of plasma LDL particles. Therefore, the LDL‐C/ApoB ratio approximately represents the cholesterol content carried by a single molecule of LDL particle. Previous studies have used LDL‐C/ApoB ratio as a surrogate indicator of LDL particle size, and found that a decreased LDL‐C/ApoB ratio was an independent risk factor for adverse cardiovascular outcomes [10, 11].

T2DM patients typically exhibit a reduction in LDL‐particle size [12, 13], which was confirmed in a study in which the LDL‐C/ApoB ratio was significantly decreased in CAD patients with underlying DM compared to those without DM [14]. In a case‐cohort study included 1,058 T2DM individuals without CAD at baseline, LDL‐C/ApoB ≤ 1.20 could predict CAD independent of ASCVD risk score [15]. However, whether the prognostic value of LDL‐C/ApoB ratio differs between diabetic and nondiabetic populations remains unclear.

In the present study, we used LDL‐C/ApoB ratio to represent LDL particle size. We aimed to investigate whether T2DM modulates the predictive value of LDL‐C/ApoB ratio on recurrent cardiovascular events in CAD population.

## 2 Results

### 2.1 Baseline Characteristics

Table 1 presents the baseline characteristics stratified by LDL‐C/ApoB levels (low level: LDL‐C/ApoB < 1.20, high level: LDL‐C/ApoB ≥ 1.20). Patients with lower LDL‐C/ApoB level were younger and more likely to be male (*p* < 0.001). The low LDL‐C/ApoB group exhibited significantly higher levels of BMI, FBG, HbA1c, TG, and creatinine, while Lp(a), HDL‐C, ApoA1 and TC were significantly lower compared to the group with high LDL‐C/ApoB (*p* < 0.05). Patients in the low LDL‐C/ApoB group had higher proportions of hypertension, dyslipidemia, smoking history, prior MI, T2DM, and usage of β‐blocker and CCB (*p* < 0.05).

**TABLE 1:** Baseline characteristics in patients stratified by groups of LDL‐C/ApoB.

Variables	Total *n* = 21,382	LDL‐C/ApoB<1.20 *n* = 10,410	LDL‐C/ApoB≥1.20 *n* = 10,972	*p*‐value
Baseline characteristics				
Age, years	59.67 ± 10.11	58.84 ± 10.25	60.45 ± 9.92	<0.001
Male	16392 (76.66)	8344 (80.15)	8048 (73.35)	<0.001
BMI, kg/m2	25.91 ± 3.23	26.13 ± 3.20	25.71 ± 3.25	<0.001
Hypertension	13655 (63.86)	6771 (65.04)	6884 (62.74)	<0.001
Dyslipidemia	16672 (77.97)	8387 (80.57)	8285 (75.51)	<0.001
Smoking history	14038 (65.65)	7090 (68.11)	6948 (63.32)	<0.001
Prior MI	5210 (24.37)	2871 (27.58)	2339 (21.32)	<0.001
Prior stroke	2696 (12.61)	1322 (12.70)	1374 (12.52)	0.713
Prior PAD	1323 (6.19)	652 (6.26)	671 (6.12)	0.675
T2DM	9360 (43.78)	4957 (47.62)	4403 (40.13)	<0.001
Family history of CAD	2506 (11.72)	1266 (12.16)	1240 (11.30)	0.053
Laboratory tests				
FBG, mmol/L	6.52 ± 2.40	6.66 ± 2.52	6.38 ± 2.26	<0.001
HbA1c, %	6.46 ± 1.23	6.53 ± 1.25	6.39 ± 1.19	<0.001
LDL‐C/Apo B	1.21 ± 0.21	1.05 ± 0.12	1.36 ± 0.15	<0.001
Apo B, g/L	0.77 ± 0.23	0.73 ± 0.21	0.80 ± 0.25	<0.001
LDL‐C, mg/dL	93.60 ± 34.83	76.86 ± 24.56	109.48 ± 35.70	<0.001
HDL‐C, mg/dL	42.79 ± 11.40	40.09 ± 10.44	45.35 ± 11.68	<0.001
Apo A1, g/L	1.36 ± 0.27	1.32 ± 0.26	1.40 ± 0.28	<0.001
TC, mg/dL	156.00 ± 40.97	140.03 ± 33.79	171.15 ± 41.43	<0.001
TG, mg/dL	129.31 [96.54–178.91]	141.71 [102.74–202.83]	120.46 [91.23–160.31]	<0.001
Lp(a), mg/L	174.18 [72.90–426.00]	169.90 [68.00–438.34]	177.70 [76.72–414.10]	0.008
CK‐MB, IU/L	11.00 [9.00–14.00]	11.00 [9.00–14.00]	11.00 [9.00–14.00]	0.09
Creatinine, µmol/L	83.01 ± 19.12	84.37 ± 20.35	81.71 ± 17.78	<0.001
LVEF, %	62.04 ± 6.85	61.97 ± 6.86	62.10 ± 6.84	0.307
Medications at discharge				
β‐blocker	18,718 (87.54)	9274 (89.09)	9444 (86.07)	<0.001
CCB	7285 (34.07)	3567 (34.27)	3718 (33.89)	0.569
Nitrate	20,488 (95.82)	9940 (95.49)	10,548 (96.14)	0.019
Antidiabetic drugs	7070 (33.1)	3699 (35.5)	3371 (30.7)	<0.001
DAPT	21,251 (99.4)	10,338 (99.3)	10,913 (99.5)	0.176

*Note*: Data were presented as mean ± SD, median with 25^th^ and 75^th^ percentile or percentages.

Abbreviations: Apo B, apolipoprotein B; BMI, body mass index; CCB, calcium channel blocker; CK‐MB, creatine kinase myocardial band; DAPT, dual antiplatelet therapy; FBG, fasting blood glucose; HbA1c, glycosylated hemoglobin A1c; HDL‐C, high‐density lipoprotein cholesterol; LDL‐C, low‐density lipoprotein cholesterol; Lp(a), lipoprotein (a); LVEF, left ventricular ejection fraction; MI, myocardial infarction; PAD, peripheral artery disease; TC, total cholesterol; TG, triglyceride.

The baseline characteristics of patients according to diabetes status are shown in **Table** **S1**. Patients with T2DM were older and more likely to be female (*p* < 0.001). Patients with T2DM had significantly higher levels of BMI, FBG, HbA1c, TG, Lp(a) and lower levels of LDL‐C/ApoB, HDL‐C, Apo A1, LVEF. Comorbidities were more common in those with T2DM, including hypertension, dyslipidemia, prior MI, prior stroke, and prior PAD. In the T2DM group, the proportion of smoking history was lower, and the proportions of β‐blocker, CCB and nitrate use were higher (*p* < 0.001). Notably, LDL‐C was slightly lower in the T2DM group than the non‐T2DM group (92.48 ±34.78 vs. 94.47 ± 34.85 mg/dL).

### 2.2 LDL‐C/ApoB Levels and Cardiovascular Outcomes

**FIGURE 1:**
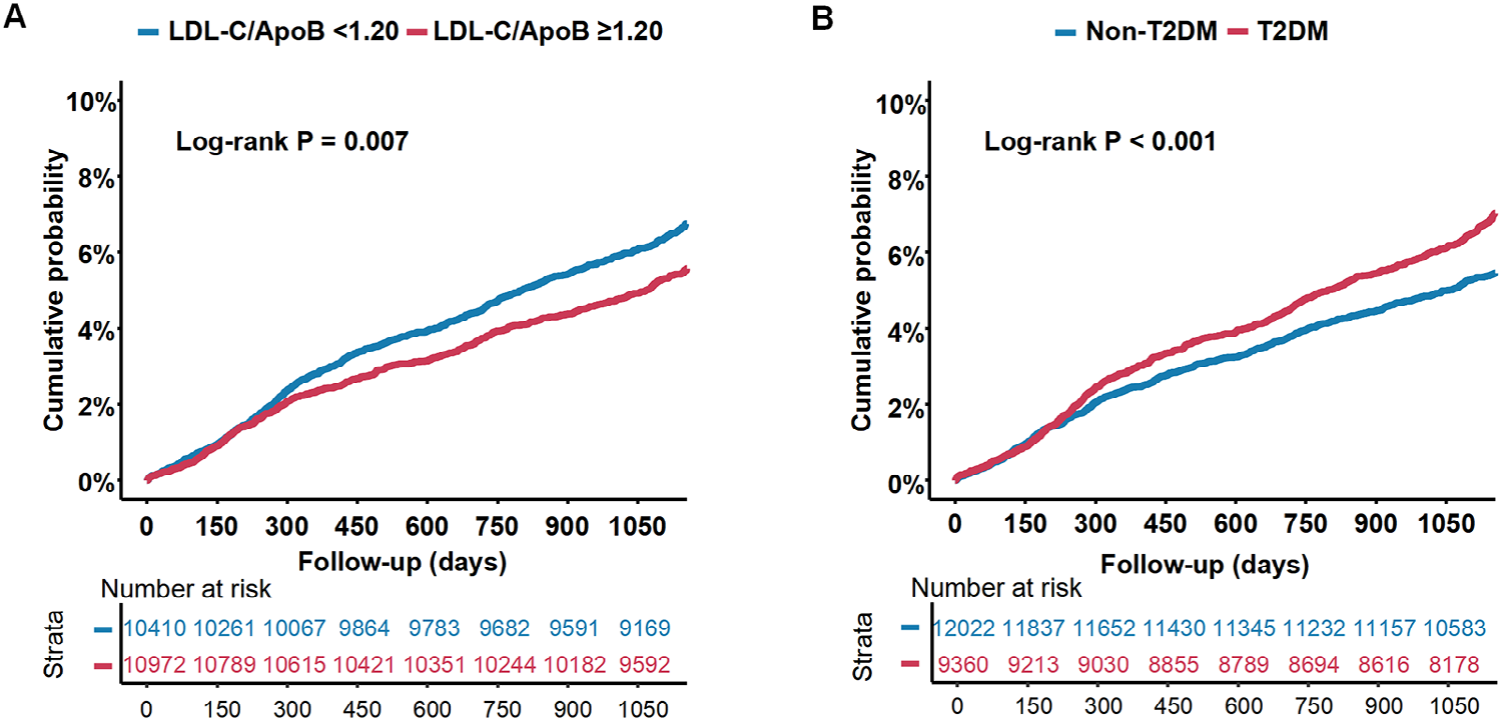
The cumulative incidence curves of MACE for patients by (A) diabetes status and (B) LDL‐C/ApoB levels. ApoB, apolipoprotein B; LDL‐C, low‐density lipoprotein cholesterol; T2DM, type 2 diabetes mellitus.

During a median follow‐up time of 3.1 (3.0–3.3) years, 1291 (6.0%) of 21,382 patients occurred MACE, including 433 (2.0%) all‐cause deaths, 170 (0.8%) non‐fatal MI, and 773 (3.6%) unplanned ischemia‐driven revascularizations. The cumulative incidence of MACE was significantly higher in patients with low LDL‐C/ApoB (<1.20) than in those with high LDL‐C/ApoB (Log‐rank p = 0.007; **Figure** **1A**). Across the whole cohort, high LDL‐C/ApoB (≥1.20) was associated with lower risk of MACE (adjusted HR: 0.88, 95% CI: 0.79–0.99, *p* = 0.032; **Table** **S2**). As shown in **Figure** **S1**, there was a nonlinear association between LDL‐C/ApoB and MACE risk in the fully adjusted model (P_nonlinear_ = 0.043). Specifically, when LDL‐C/ApoB was below 1.20, MACE risk decreased with increasing LDL‐C/ApoB.

### 2.3 LDL‐C/ApoB Levels, T2DM, and Cardiovascular Outcomes

Then, we divided the whole cohort according to the absence or presence of T2DM. A total of 9360 (43.78%) patients had T2DM at baseline, and the cumulative incidence of MACE was significantly higher in patients with T2DM than in those without T2DM (Log‐rank p<0.001; **Figure** **1B**).

In the T2DM cohort, high LDL‐C/ApoB was associated with lower risks of MACE (adjusted HR: 0.81, 95% CI: 0.69–0.96, *p* = 0.010; **Table** **2**). As shown in **Figure** **S2**, there was a linear association between LDL‐C/ApoB and MACE risk in the fully adjusted model (P_nonlinear_ > 0.05). A stepwise increase of 0.1 unit in LDL‐C/ApoB ratio was associated with a 4% decrease for MACE in the T2DM subgroup. However, there was no significant association between LDL‐C/ApoB and MACE in the non‐T2DM cohort whether as a categorical variable or a continuous variable (**Table** **2**, **Figure** **S3**). Notably, a significant interaction was observed between the LDL‐C/ApoB ratio (both as a continuous and a categorical variable) and T2DM regarding the risk of MACE (*p* for interaction < 0.05; **Table** **2**).

**TABLE 2:** Relation of the LDL‐C/ApoB levels and MACE in patients with or without diabetes.

Variables	Univariate analysis		Multivariate analysis^a^	
	HR (95% CI)	*p*‐value	HR (95% CI)	*p*‐value
**T2DM cohort**				
LDL‐C/ApoB<1.20	Reference	—	Reference	—
LDL‐C/ApoB≥1.20	0.78 (0.67–0.92)	0.002	0.81 (0.69–0.96)	0.010
LDL‐C/ApoB per 0.1‐unit increase	0.93 (0.90–0.97)	<0.001	0.95 (0.91–0.99)	0.013
**Non‐T2DM cohort**				
LDL‐C/ApoB<1.20	Reference	—	Reference	—
LDL‐C/ApoB≥1.20	0.93 (0.79–1.09)	0.360	0.95 (0.81–1.11)	0.522
LDL‐C/ApoB per 0.1‐unit increase	0.99 (0.96–1.03)	0.761	0.99 (0.95–1.03)	0.627

Abbreviations: ApoB, apolipoprotein B; CI, confidence interval; HR, hazard ratio; LDL‐C, low‐density lipoprotein cholesterol; MACE, major adverse cardiovascular events; T2DM, type 2 diabetes mellitus.

^a^ The model was adjusted for age, sex, BMI, smoking history, hypertension, prior MI, family history of CAD, LVEF, serum creatinine, HDL‐C, TG, HbA1c, duration of diabetes, Syntax score and beta‐blocker.

^b^ P for interaction for the risk of MACE: continuous LDL‐C/ApoB and T2DM = 0.024; categorical groups of LDL‐C/ApoB (<1.20 or ≥1.20) and T2DM = 0.048.

**FIGURE 2:**
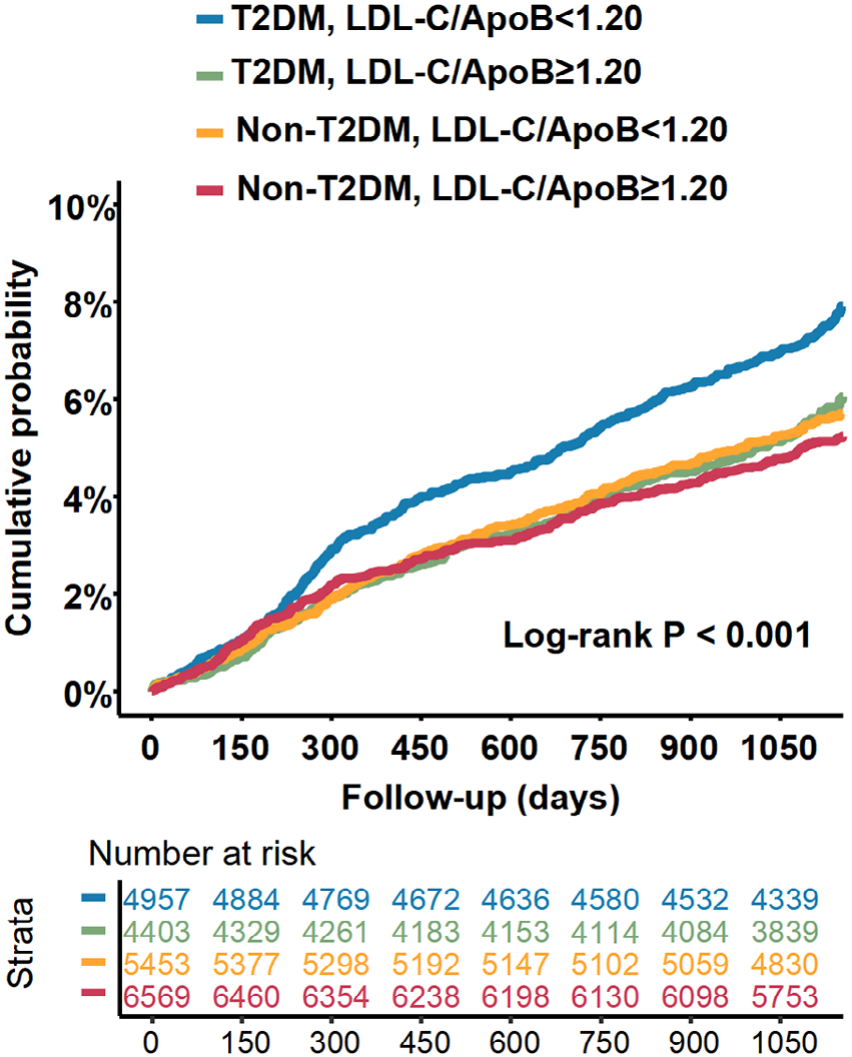
The cumulative incidence curves of MACE for patients by both diabetes status and LDL‐C/ApoB levels. ApoB, apolipoprotein B; LDL‐C, low‐density lipoprotein cholesterol; T2DM, type 2 diabetes mellitus.

Furthermore, we evaluated the prognostic value of LDL‐C/ApoB levels plus diabetes status. Kaplan–Meier analysis shows that according to the subgroups by both status, patients with T2DM plus low LDL‐C/ApoB had the highest incidence of MACE (Log‐rank P < 0.001, **Figure** **2**). Multivariable Cox regression models identified patients with T2DM plus low LDL‐C/ApoB had the highest MACE risk (P for trend <0.001) among the four groups. Compared to the reference group of T2DM plus low LDL‐C/ApoB level, the groups of T2DM plus high‐level, non‐T2DM plus low‐level, and non‐T2DM plus high‐level LDL‐C/ApoB had lower risks of MACE in both unadjusted and adjusted models (adjusted HR: 0.79, 95% CI: 0.67–0.93, *p* = 0.003; adjusted HR: 0.78, 95% CI: 0.67–0.91, *p* = 0.001; adjusted HR: 0.75, 95% CI: 0.65–0.87, *p* < 0.001; respectively; **Table** **3**).

**TABLE 3:** Predictive value of LDL‐C/ApoB levels and diabetes status for MACE.

Variables^a^	Events/subjects	Univariate analysis		Multivariate analysis ^b^, ^c^	
		HR (95% CI)	*p* value	HR (95% CI)	*p* value
Low, T2DM	379/4957 (7.6)	Reference	—	Reference	—
High, T2DM	262/4403 (6.0)	0.78 (0.67–0.91)	0.002	0.79 (0.67–0.93)	0.003
Low, non‐T2DM	301/5453 (5.5)	0.72 (0.62–0.83)	<0.001	0.78 (0.67–0.91)	0.001
High, non‐T2DM	349/6569 (5.3)	0.70 (0.61–0.81)	<0.001	0.75 (0.65–0.87)	<0.001

Abbreviations: ApoB, apolipoprotein B; CI, confidence interval; HR, hazard ratio; LDL‐C, low‐density lipoprotein cholesterol; MACE, major adverse cardiovascular events; T2DM, type 2 diabetes mellitus.

^a^ Low: LDL‐C/ApoB <1.20; High: LDL‐C/ApoB ≥1.20.

^b^ The model was adjusted for age, sex, BMI, smoking history, hypertension, prior MI, family history of CAD, LVEF, serum creatinine, HDL‐C, TG, HbA1c, duration of diabetes, Syntax score and beta‐blocker.

^c^ P for trend for the risk of MACE < 0.001.

**FIGURE 3:**
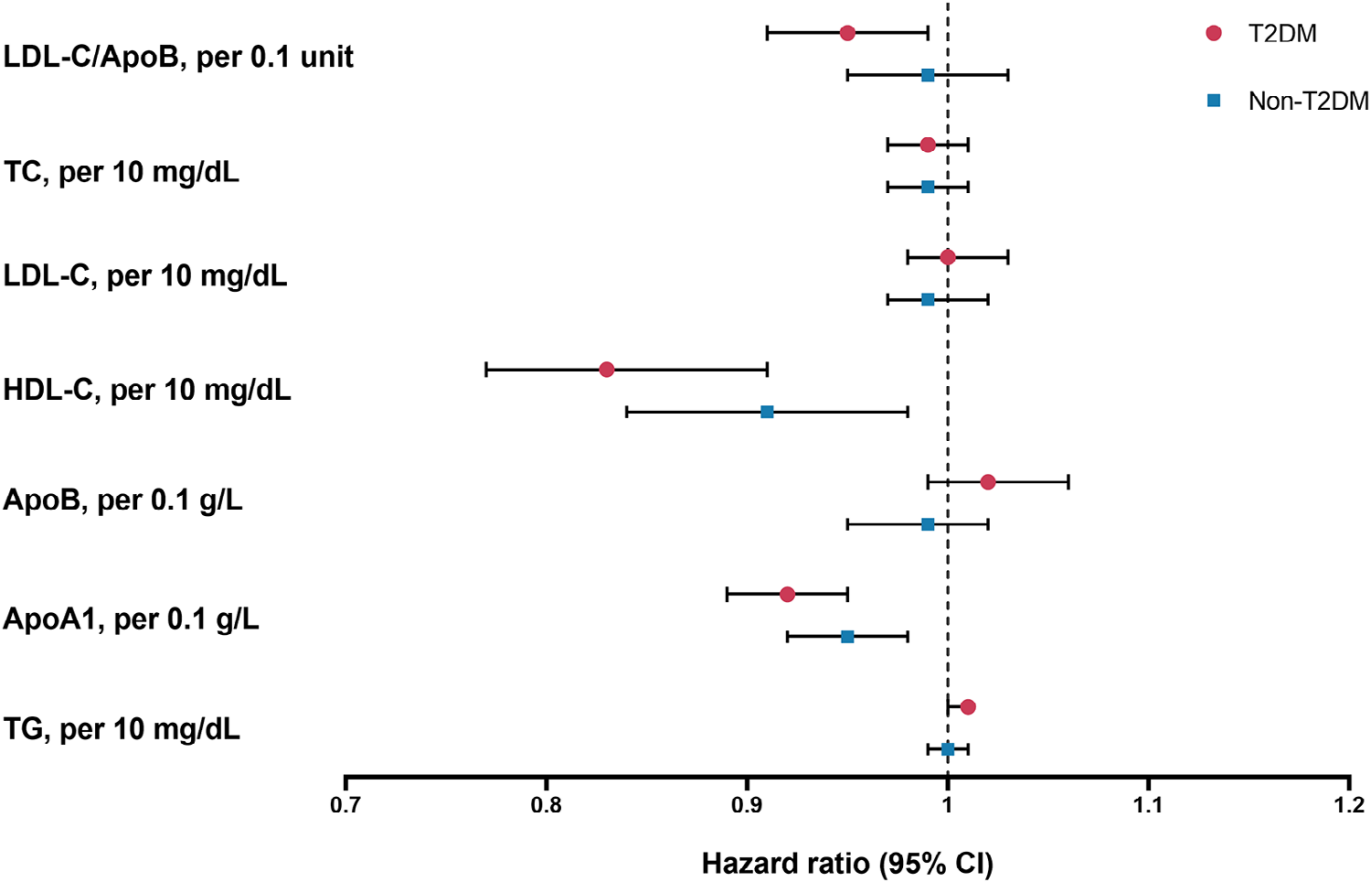
The predictive value for MACE of traditional lipid parameters. Each parameter was included in the model as an increasing continuous variable. The model was adjusted for age, sex, BMI, smoking history, hypertension, prior MI, family history of CAD, LVEF, serum creatinine, HDL‐C and TG. ApoA1, apolipoprotein A1; ApoB, apolipoprotein B; CI, confidential interval; HDL‐C, high‐density lipoprotein cholesterol; LDL‐C, low‐density lipoprotein cholesterol; T2DM, type 2 diabetes mellitus; TC, total cholesterol; TG, triglyceride.

We also investigated the traditional lipid parameters as predictors of MACE in Cox regression models (**Table** **S3**). As shown in **Figure** **3**, no significant association was identified between MACE risk and LDL‐C or ApoB when considered individually in the T2DM subgroup. HDL‐C and ApoA1 were inversely associated with MACE risk both in the T2DM and non‐T2DM groups.

### 2.4 Subgroup Analysis

To further investigate the association of LDL‐C/ApoB ratio with MACE risk, we conducted stratified analyses by age, sex, clinical presentation, CKD and hypertension. Results were consistent across different subgroups. No significant interaction between LDL‐C/ApoB ratio and age, sex, clinical presentation, CKD or hypertension was observed (*p* for interaction >0.05, **Table** **S4**).

## 3 Discussion

In this large‐scale, prospective cohort study, we investigated, for the first time, the prognostic value of the LDL‐C/ApoB ratio in statin‐treated CAD patients with or without T2DM. The major findings of the present study included the following aspects: (1) In statin‐treated CAD patients, a low LDL‐C/ApoB level (<1.20) was independently associated with a worse prognosis in the overall population; (2) Stratification by diabetes status revealed that low levels of LDL‐C/ApoB predicted a worse prognosis specifically in patients with T2DM, but not in those without T2DM; (3) Patients with T2DM and low LDL‐C/ApoB levels had the highest risks for MACE compared to those in the other three groups; (4) No significant association was identified between MACE risk and LDL‐C or ApoB when considered individually in the T2DM subgroup. For the first time, we compared the prognostic value of LDL‐C/ApoB between statin‐treated CAD patients with or without T2DM, and evaluated the synergetic effect of LDL‐C/ApoB and T2DM in statin‐treated CAD patients.

Quantities of studies have proved the relationship between LDL particle size and cardiovascular outcomes. The 13‐year follow‐up data from the Québec Cardiovascular Study showed that estimated cholesterol levels in the small LDL subfraction (<25.5 nm) were strongly and independently associated with the long‐term risk of ischemic heart disease, and elevated LDL‐C levels in the large LDL subfraction (>26.0 nm) did not confer an increased cardiovascular risk [16]. In the Atherosclerosis Risk In Communities (ARIC) study, elevated sdLDL‐C levels can predict incident CAD, even in individuals considered to be at low cardiovascular risk based on the LDL‐C levels [17]. As an indirect measure of LDL‐particle size, Drexel et al. demonstrated that the LDL‐C/ApoB ratio was an independent predictor for MACE in patients with established ASCVD [11]. Here, we investigated, for the first time, the prognostic value of the LDL‐C/ApoB ratio in statin‐treated CAD patients, and the results likewise revealed a significant association between LDL‐C/ApoB and cardiovascular outcomes in the fully adjusted model.

Dyslipidemia is more common in the T2DM population than non‐T2DM population, which is a crucial reason for the increased cardiovascular risks in individuals with T2DM [18]. However, plasma LDL‐C levels, the primary target of lipid‐lowering therapies, are usually normal‐to‐mildly elevated in the T2DM population [18, 19], which cannot reflect the residual cholesterol risk adequately. In the present study, LDL‐C levels were slightly lower in the T2DM group (92.48 ± 34.78 vs. 94.47 ± 34.85 mg/dL), but this was not clinically significant. This may be because all patients included in this study were on statin therapy, and patients in the T2DM group may have received more stringent risk factor management. Besides, similar to several recent studies [20, 21, 22], we found that in patients with CAD who received lipid‐lowering treatment, LDL‐C might not fully reflect the residual cholesterol risk. Thus, in addition to the quantitative abnormalities, qualitative abnormalities of plasma lipid profiles also play an important role in diabetes‐related dyslipidemia. Several studies demonstrated that patients with diabetes tended to have a smaller mean LDL particle diameter and a higher proportion of sdLDL to total LDL compared to those without diabetes [23, 24, 25], especially in those with CAD [14].

To date, there is limited research that has compared the prognostic value of LDL‐particle size in patients with or without T2DM. Jin et al. demonstrated that elevated sdLDL level was associated with increased risk of MACE in patients with CAD and DM, while there is no association in patients with prediabetes and normal glycemia regulation [26]. They found a significant interaction between sdLDL and diabetic status, aligning with our observation that the association is more pronounced in T2DM. Unlike them, we used LDL‐C/ApoB, a cheaper and simpler method, to indirectly represent LDL particle size. Both of our studies indicated that LDL‐particle size may have more clinical significance in the population with diabetes and should be more widely evaluated in the management of diabetes. Conversely, some studies have also reported a significant predictive value of LDL‐particle size in non‐diabetic cohorts as well [10, 27]. These discrepancies may be explained by differences in study populations: our cohort specifically consisted of statin‐treated patients undergoing secondary prevention, in contrast to the primary prevention populations included in the prior studies.

The pathological mechanisms that lead to a reduction in the diameter of LDL particles in T2DM may be explained as follows: (1) Insulin resistance stimulates the liver to produce more very low‐density lipoprotein (VLDL) particles, which are the precursor to sdLDL particles [18]; (2) Decreased activity of lipoprotein lipase leads to a reduced conversion of VLDL to LDL [28, 29]; (3) As VLDL and its remnants accumulate, cholesterol‐rich LDLs transfer into TG‐rich LDLs via the action of cholesteryl ester transfer protein (CETP); TG‐rich LDLs become preferred substrates for hepatic lipase, which further breaks down the TG within the LDL core, leading to the formation of sdLDL [12].

Although the clinical significance of LDL‐particle size has been illustrated in abundant studies, how to improve the lipid profile of patients with T2DM still needs more explorations [30]. According to the latest 2023 ESC Guidelines for the management of cardiovascular disease in patients with diabetes, sodium‐glucose co‐transporter‐2 inhibitors (SGLT2i) and glucagon‐like peptide‐1 receptor agonists (GLP‐1RAs) were recommended to reduce cardiovascular risk independent of glucose control in patients with T2DM and ASCVD [19]. The question has been raised whether these novel antidiabetic therapies could affect LDL subfractions in patients with T2DM [31, 32]. Hayashi et al. conducted a randomized trial to compare the effect of dapagliflozin with sitagliptin on LDL and HDL subfractions in patients with T2DM [33]. Dapagliflozin administration did not significantly alter concentrations of LDL‐C and apoB, while decreased sdLDL cholesterol by 20% and increased lbLDL cholesterol by 18%. In a randomized, double‐blind, placebo‐controlled, cross‐over trial, liraglutide combined with metformin significantly suppressed sdLDL fraction by 16% in patients with CAD and T2DM on stable statin therapy [34]. Another real‐world prospective study on patients with T2DM also confirmed that the vascular benefit of liraglutide was associated with reductions in sdLDL, independent of the effect of glycemic control and body weight reduction [35]. In summary, novel antidiabetic medications may improve LDL subfractions, thus contributing to the cardiovascular protection effects they exhibit.

This is the first study to investigate the prognostic value of LDL‐C/ApoB in statin‐treated CAD patients with T2DM, suggesting that this ratio can serve as an independent predictor for residual cholesterol risk in the management of such patients. However, the current study still has several limitations. First, although we included a population of patients who had received statin therapy at baseline to circumvent the drug's effect on lipid levels, we lacked data on statin intensity. Second, it is worth noting that PCSK9 inhibitors and novel oral hypoglycemic agents were not widely used in China at the time of this study, and the effect of these drugs still need more research. Third, due to the natural defects of cohort studies, undetected confounders may result in potential bias. Fourth, this is a single‐center study of the Chinese population, which lacks diversity and may affect the generalizability of the findings. Fifth, as an observational study, the present analysis can identify associations but cannot establish causality; further randomized controlled trials are warranted to confirm any potential causal relationships.

## 4 Conclusion

Our data demonstrated that in CAD patients who had received statin therapy, LDL‐C/ApoB ratio was related to adverse prognosis only in those with T2DM. Among patients with CAD and T2DM who are receiving statin treatment, LDL‐C/ApoB may be a better indicator of residual cholesterol risk than LDL‐C.

## 5 Materials and Methods

### 5.1 Study Design and Population

**FIGURE 4:**
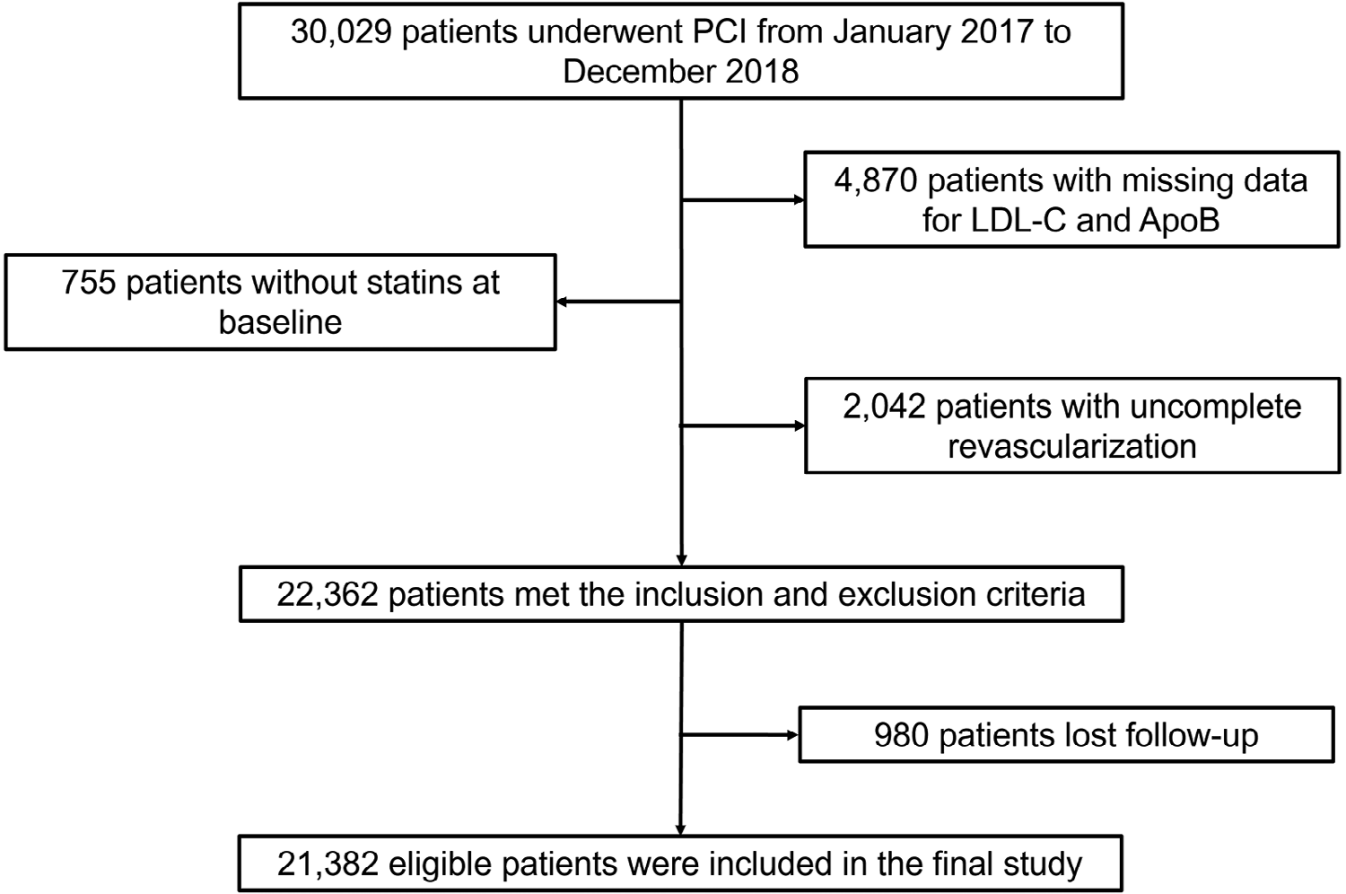
Study flowchart. ApoB, apolipoprotein B; LDL‐C, low‐density lipoprotein cholesterol; PCI, percutaneous coronary intervention.

The study design of the present study was a large‐scale, single‐center, observational cohort study. From January 2017 to December 2018, 30,029 consecutive CAD patients who underwent percutaneous coronary intervention (PCI) at Fuwai Hospital were initially recruited (**Figure** **4**). CAD was defined as a documented history of myocardial infarction (MI), PCI, coronary artery bypass grafting (CABG), or angiographic evidence of ≥50% coronary stenosis. Further exclusion of the initial population is based on the following criteria: (1) patients with missing data for LDL‐C or ApoB; (2) patients without statins at baseline; (3) patients with uncomplete revascularization; (4) patients lost to follow‐up. Ultimately, a total of 21,382 eligible patients were included in the final analysis. According to the previous studies [9], an LDL‐C/ApoB value of 1.2, corresponding to an LDL diameter of 25.5 nm, is proposed as the optimal cut‐off value to distinguish sdLDL from lbLDL. Thus, the population was further divided into low LDL‐C/ApoB (<1.2) and high LDL‐C/ApoB (≥1.2) groups.

All participants were followed up routinely every 6 months via clinic visits or telephone interviews by dedicated research coordinators who were blinded to the study objectives [36]. The primary endpoint was major adverse cardiac events (MACE), a composite of all‐cause mortality, non‐fatal myocardial infarction (MI), and unplanned ischemia‐driven revascularization. All deaths were verified using medical records, death certificates, and reports from family members. Non‐fatal MI required elevated cardiac troponins alongside typical ischemic symptoms or electrocardiogram changes. Ischemia‐driven revascularization was defined as any repeat PCI or CABG driven by an ischemic event. All potential endpoint events were first reviewed and confirmed independently by two experienced cardiologists blinded to the study data. In case of disagreement, a third senior cardiologist was consulted to reach a final adjudication [37].

The study was conducted in accordance with the Declaration of Helsinki. Approval was obtained from the Institutional Review Board of Fuwai Hospital (Beijing, China). All participants provided written informed consent.

### 5.2 Clinical Assessment

Baseline data, including demographics, comorbidities, family history, and medications, were collected via self‐reported medical history. DM was diagnosed according to American Diabetes Association criteria, based on elevated fasting blood glucose (≥7.0 mmol/L), 2‐h plasma glucose (≥11.1 mmol/L), HbA1c (≥6.5%), or use of hypoglycemic drugs. Hypertension was defined as blood pressure ≥140/90 mmHg or use of antihypertensives. Body mass index (BMI) was derived as weight (kg) / height (m)^2^.

### 5.3 Measurement of Laboratory Tests

Fasting blood samples (≥12 h) were collected on admission and analyzed in the Fuwai hospital's clinical chemistry department. Routine biochemistry parameters—including total cholesterol (TC), high‐density lipoprotein‐cholesterol (HDL‐C), LDL‐C, triglyceride (TG), serum creatinine, and CK‐MBwere measured enzymatically using an automated analyzer (Hitachi 7150, Japan). ApoB levels were determined by an immunoturbidimetric assay (Tina‐quant, Roche Diagnostics). LDL particle size was reflected indirectly by LDL‐C/ApoB ratio. Fasting blood glucose (FBG) was analyzed with the enzymatic hexokinase method, and HbA1c was quantified using a Tosoh Automated Analyzer (HLC‐723G8, Tokyo, Japan). Left ventricular ejection fraction (LVEF) was calculated from two‐dimensional echocardiography using the modified Simpson's rule.

### 5.4 Statistical Analyses

Continuous variables are presented as mean ± standard deviation or median (interquartile range), as appropriate, while categorical variables are summarized as counts (percentages). For comparisons of baseline characteristics across groups, the following tests were applied: Student's *t*‐test or the Mann–Whitney *U* test for continuous variables, and the Chi‐square test or Fisher's exact test for categorical variables, depending on data distribution and sample size.

To assess associations, univariable and multivariable Cox proportional hazards models were constructed to estimate hazard ratios (HRs) with 95% confidence intervals (CIs). The multivariable models adjusted for age, sex, BMI, smoking history, hypertension, prior MI, family history of CAD, LVEF, serum creatinine, HDL‐C, TG, HbA1c, duration of diabetes, Syntax score and beta‐blocker. The proportional hazards assumption was tested using Schoenfeld residuals. To assess effect modification, a multiplicative interaction term (T2DM status × LDL‐C/ApoB ratio) was incorporated into the Cox regression models. The linearity assumption for the LDL‐C/ApoB ratio was evaluated with restricted cubic spline (RCS) analysis. Time‐to‐event outcomes were depicted using Kaplan–Meier (KM) curves, and inter‐group differences were compared with the log‐rank test. All analyses were conducted in R (version 4.1.0; R Foundation), with a two‐tailed *p* value < 0.05 deemed statistically significant.

## Author Contributions

X.B., J.H., G.G., and K.D. contributed to the study design and interpretation. S.Y., R.Z., and C.S. were responsible for data collection and analysis. J.H. and X.B. drafted the initial manuscript, which was then critically revised by G.G. and K.D. All authors read and approved the final manuscript.

## Funding

This work was supported by the Noncommunicable Chronic Diseases‐National Science and Technology Major Project (grant no.: 2025ZD0548200, 2023ZD0513900) and the Beijing Health Promotion Association (grant no.: T2022‐ZX022).

## Conflicts of Interest

The authors declare no conflicts of interest.

## Ethics Statement

The study protocol was approved by the Institutional Review Board of Fuwai Hospital (2016‐847) and was conducted in compliance with the Declaration of Helsinki. All participants provided written informed consent.

## Supporting information




**Supporting File**: mco270919‐sup‐0001‐SuppMat.docx.

## Data Availability

The datasets used and analyzed during the current study are available from the corresponding author on reasonable request.
