# Supplementary material for: Type 2 Diabetes Mellitus Modifies the Prognostic Value of LDL Particle Size in Statin‐treated Patients With Coronary Artery Disease: A Large‐scale Cohort Study
Source: MedComm (2020). 2026 Aug 26;7(9):e70919. doi: 10.1002/mco2.70919 (PMC13507696; doi:10.1002/mco2.70919)
Supplement: Supplementary file 1 [file MCO2-7-e70919-s001.docx]

**Supplementary Materials**

**Type 2 diabetes mellitus modifies the prognostic value of LDL particle size in statin-treated patients with coronary artery disease: a large-scale cohort study**

Xiaohui Bian, MD^1,2,3#^, Jining He, MD^1,2,3#^, Chenxi Song, MD^1,2,3^, Rui Zhang, MD^1,2,3^, Sheng Yuan, MD^1,2,3^, Guofeng Gao, MD^3*^, Kefei Dou, MD, PhD^1,2,3,4*^

^1^State Key Laboratory of Cardiovascular Disease, Beijing, China; ^2^Cardiometabolic Medicine Center, Fuwai Hospital, National Center for Cardiovascular Diseases, Chinese Academy of Medical Sciences and Peking Union Medical College, Beijing, China; ^3^Department of Cardiology, Fuwai Hospital, National Center for Cardiovascular Diseases, Chinese Academy of Medical Sciences and Peking Union Medical College, Beijing, China; ^4^National Clinical Research Center for Cardiovascular Diseases, Beijing, China;

#Xiaohui Bian and Jining He equally contributed to this work

*Address for correspondence:

Kefei Dou, Cardiometabolic Medicine Center, National Clinical Research Center for Cardiovascular Diseases, State Key Laboratory of Cardiovascular Disease, Fuwai Hospital, National Center for Cardiovascular Diseases, Chinese Academy of Medical Sciences and Peking Union Medical College, A 167 Beilishi Rd, Xicheng District, Beijing 100037, China. Email: drdoukefei@126.com.

AND

Guofeng Gao, Department of Cardiology, Fuwai Hospital, National Center for Cardiovascular Diseases, Chinese Academy of Medical Sciences and Peking Union Medical College, A 167 Beilishi Rd, Xicheng District, Beijing 100037, China. Email: ggf03@163.com.

**Table S1 Baseline characteristics in patients with or without T2DM**

| **Variables** | **Total**  **n=21382** | **T2DM**  **n=9360** | **Non-T2DM**  **n=12022** | **P value** |
| --- | --- | --- | --- | --- |
| Baseline characteristics | | | | |
| Age, years | 59.67±10.11 | 60.74±9.64 | 58.83±10.39 | <0.001 |
| Male | 16392 (76.66) | 6941 (74.16) | 9451 (78.61) | <0.001 |
| BMI, kg/m2 | 25.91±3.23 | 26.25±3.22 | 25.65±3.21 | <0.001 |
| Hypertension | 13655 (63.86) | 6495 (69.39) | 7160 (59.56) | <0.001 |
| Dyslipidemia | 16672 (77.97) | 7603 (81.23) | 9069 (75.44) | <0.001 |
| Smoking history | 14038 (65.65) | 6011 (64.22) | 8027 (66.77) | <0.001 |
| Prior MI | 5210 (24.37) | 2412 (25.77) | 2798 (23.27) | <0.001 |
| Prior stroke | 2696 (12.61) | 1417 (15.14) | 1279 (10.64) | <0.001 |
| Prior PAD | 1323 (6.19) | 691 (7.38) | 632 (5.26) | <0.001 |
| Family history of CAD | 2506 (11.72) | 1071 (11.44) | 1435 (11.94) | 0.274 |
| Laboratory tests | | | | |
| FBG, mmol/L | 6.52±2.40 | 8.13±2.83 | 5.26±0.61 | <0.001 |
| HbA1c, % | 6.46±1.23 | 7.41±1.29 | 5.72±0.35 | <0.001 |
| LDL-C/Apo B | 1.21±0.21 | 1.19±0.21 | 1.23±0.20 | <0.001 |
| Apo B, g/L | 0.77±0.23 | 0.77±0.23 | 0.76±0.23 | 0.056 |
| LDL-C, mg/dL | 93.60±34.83 | 92.48±34.78 | 94.47±34.85 | <0.001 |
| HDL-C, mg/dL | 42.79±11.40 | 41.53±11.01 | 43.77±11.61 | <0.001 |
| Apo A1, g/L | 1.36±0.27 | 1.34±0.27 | 1.37±0.27 | <0.001 |
| TC, mg/dL | 156.00±40.97 | 154.87±41.38 | 156.87±40.63 | <0.001 |
| TG, mg/dL | 129.31 [96.54-178.91] | 133.74 [100.08-189.54] | 125.77 [93.88-170.94] | <0.001 |
| Lp(a), mg/L | 174.18 [72.90-426.00] | 182.23 [76.40-437.00] | 164.39 [67.57-412.02] | <0.001 |
| CK-MB, IU/L | 11.00 [9.00-14.00] | 11.00 [9.00-15.00] | 11.00 [8.00-14.00] | <0.001 |
| Creatinine, μmol/L | 83.01±19.12 | 83.47±21.33 | 82.64±17.18 | 0.002 |
| LVEF, % | 62.04±6.85 | 61.55±7.19 | 62.42±6.55 | <0.001 |
| Medications at discharge | | | | |
| β-blocker | 18718 (87.54) | 8384 (89.57) | 10334 (85.96) | <0.001 |
| CCB | 7285 (34.07) | 3488 (37.26) | 3797 (31.58) | <0.001 |
| Nitrate | 20488 (95.82) | 9003 (96.19) | 11485 (95.53) | 0.020 |

Data were presented as mean ± SD, median with 25^th^ and 75^th^ percentile or percentages.

BMI, body mass index; MI, myocardial infarction; PAD, peripheral artery disease; FBG, fasting blood glucose; HbA1c, glycosylated hemoglobin A1c; LDL-C, low-density lipoprotein cholesterol; Apo B, apolipoprotein B; HDL-C, high-density lipoprotein cholesterol; TC, total cholesterol; TG, triglyceride; Lp(a), lipoprotein (a); CK-MB, creatine kinase myocardial band; LVEF, left ventricular ejection fraction; CCB, calcium channel blocker

**Table S2 Relation of LDL-C/ApoB and MACE in the whole cohort**

| **LDL-C/ApoB** | **Univariable analysis** | | **Multivariable analysis*** | |
| --- | --- | --- | --- | --- |
|  | **HR (95% CI)** | **P value** | **HR (95% CI)** | **P value** |
| Categorical | | | | |
| Low level (<1.20) | Reference | - | Reference | - |
| High level (≥1.20) | 0.86 (0.77-0.96) | 0.007 | 0.88 (0.79-0.99) | 0.032 |
| Continuous | | | | |
| Per 0.1-unit increase | 0.96 (0.94-0.99) | 0.005 | 0.97 (0.94-0.99) | 0.036 |

*The model was adjusted for age, sex, BMI, smoking history, hypertension, prior MI, family history of CAD, LVEF, serum creatinine, HDL-C, TG, HbA1c, duration of diabetes, Syntax score and beta-blocker.

HR, hazard ratio; CI, confidence interval; other abbreviations as in Table S1.

**Table S3 Relation of traditional lipid parameters and MACE**

|  | **Univariable analysis** | | **Multivariable analysis*** | |
| --- | --- | --- | --- | --- |
|  | **HR (95% CI)** | **P value** | **HR (95% CI)** | **P value** |
| T2DM | | | | |
| TC, per 10 mg/dL | 0.99 (0.97-1.01) | 0.484 | 0.99 (0.97-1.01) | 0.521 |
| LDL-C, per 10 mg/dL | 0.99 (0.97-1.02) | 0.546 | 1.00 (0.98-1.03) | 0.878 |
| HDL-C, per 10 mg/dL | 0.85 (0.78-0.91) | <0.001 | 0.83 (0.77-0.91) | <0.001 |
| Apo B, per 0.1 g/L | 1.02 (0.98-1.05) | 0.321 | 1.02 (0.99-1.06) | 0.212 |
| Apo A1, per 0.1 g/L | 0.92 (0.89-0.95) | <0.001 | 0.92 (0.89-0.95) | <0.001 |
| TG, per 10 mg/dL | 1.00 (1.00-1.01) | 0.202 | 1.01 (1.00-1.01) | 0.017 |
| Non-T2DM | | | | |
| TC, per 10 mg/dL | 0.99 (0.97-1.00) | 0.130 | 0.99 (0.97-1.01) | 0.305 |
| LDL-C, per 10 mg/dL | 0.99 (0.97-1.01) | 0.269 | 0.99 (0.97-1.02) | 0.475 |
| HDL-C, per 10 mg/dL | 0.94 (0.87-1.00) | 0.056 | 0.91 (0.84-0.98) | 0.013 |
| Apo B, per 0.1 g/L | 0.98 (0.94-1.01) | 0.163 | 0.99 (0.95-1.02) | 0.475 |
| Apo A1, per 0.1 g/L | 0.96 (0.93-0.99) | 0.003 | 0.95 (0.92-0.98) | 0.002 |
| TG, per 10 mg/dL | 0.99 (0.98-1.00) | 0.205 | 1.00 (0.99-1.01) | 0.933 |

*The model was adjusted for age, sex, BMI, smoking history, hypertension, prior MI, family history of CAD, LVEF, serum creatinine, HDL-C, TG, HbA1c, duration of diabetes, Syntax score and beta-blocker.

**Table S4 Subgroup analysis for MACE as the adjusted model stratified according to LDL-C/ApoB levels**

| **Variables** | **LDL-C/ApoB<1.20** | **LDL-C/ApoB≥1.20*** | **P for interaction*** |
| --- | --- | --- | --- |
| **Sex** | | | **0.270** |
| Male | 1.0 (Reference) | 0.92 (0.81-1.05) |  |
| Female | 1.0 (Reference) | 0.77 (0.61-0.98) |  |
| **Clinical presentation** | | | **0.518** |
| ACS | 1.0 (Reference) | 0.87 (0.75-1.00) |  |
| CCS | 1.0 (Reference) | 0.92 (0.76-1.12) |  |
| **CKD** | | | **0.128** |
| No | 1.0 (Reference) | 0.87 (0.78-0.98) |  |
| Yes | 1.0 (Reference) | 1.43 (0.82-2.50) |  |
| **Hypertension** | | | **0.344** |
| No | 1.0 (Reference) | 0.92 (0.76-1.12) |  |
| Yes | 1.0 (Reference) | 0.86 (0.75-0.99) |  |

*The model was adjusted for age, sex, BMI, smoking history, hypertension, prior MI, family history of CAD, LVEF, serum creatinine, HDL-C, TG, HbA1c, duration of diabetes, Syntax score and beta-blocker.

**Figure S1 Restricted cubic spline analysis for association of the LDL-C/ApoB and risk of MACE across the entire cohort in (A)unadjusted and (B)adjusted models.**

The model was adjusted for age, sex, BMI, smoking history, hypertension, prior MI, family history of CAD, LVEF, serum creatinine, HDL-C, TG, HbA1c, duration of diabetes, Syntax score and beta-blocker.

**Figure S2 Restricted cubic spline analysis for association of the LDL-C/ApoB and risk of MACE across T2DM subgroup in (A)unadjusted and (B)adjusted models.**

The model was adjusted for age, sex, BMI, smoking history, hypertension, prior MI, family history of CAD, LVEF, serum creatinine, HDL-C, TG, HbA1c, duration of diabetes, Syntax score and beta-blocker.

**Figure S3 Restricted cubic spline analysis for association of the LDL-C/ApoB and risk of MACE across non-T2DM subgroup in (A)unadjusted and (B)adjusted models.**

The model was adjusted for age, sex, BMI, smoking history, hypertension, prior MI, family history of CAD, LVEF, serum creatinine, HDL-C, TG, HbA1c, duration of diabetes, Syntax score and beta-blocker.
